# Mechanisms and targeted intervention strategies of macrophage mitochondrial quality control imbalance in the progression of atherosclerosis

**DOI:** 10.3389/fphys.2026.1975785

**Published:** 2026-10-22

**Authors:** Guo Zhili, Zhu Xiaoyu

**Affiliations:** 1 Jiaxing Hospital of Traditional Chinese Medicine, Jiaxing University, Jiaxing, Zhejiang, China; 2 Zhejiang Chinese Medical University, Hangzhou, China

**Keywords:** atherosclerosis, macrophage polarization, macrophages, mitochondrial DNA, Mitochondrial quality control, mitophagy, NLRP3 inflammasome, reactive oxygen species

## Abstract

Atherosclerosis (AS) is a vascular disease characterized by lipid accumulation, chronic inflammation, and plaque instability, which can lead to severe cardiovascular events, such as myocardial infarction and stroke. Macrophages are the predominant immune cells within plaques and form foam cells upon uptake of oxidized low-density lipoprotein, a critical driver of AS progression. Recent studies have confirmed that imbalance of the mitochondrial quality control (MQC) system participates in the initiation and progression of AS; however, the imbalance in macrophage MQC as an upstream regulatory mechanism has not yet been systematically elucidated. MQC encompasses mitophagy, fusion-fission balance, mitochondrial biogenesis, and mitochondrial DNA (mtDNA) homeostasis. This imbalance can result in impaired clearance of damaged mitochondria, dysfunction of the electron transport chain, and excessive production of reactive oxygen species (ROS), which in turn activate the NLRP3 inflammasome and trigger inflammation and apoptosis. Concurrently, mitochondrial metabolic reprogramming can promote macrophage polarization toward the pro-inflammatory M1 phenotype while suppressing the anti-inflammatory M2 phenotype, thereby exacerbating lipid deposition and inflammatory amplification. Plaque macrophages are, however, heterogeneous: TREM2-high foamy macrophages are typically less inflammatory and metabolically distinct from adjacent non-foamy macrophages, cautioning against a purely linear glycolysis-M1-foam-cell sequence. Current research predominantly focuses on MQC imbalance, whereas a systematic integration of how imbalances in the MQC network mediate macrophage inflammation, foam cell formation, and plaque progression remains lacking. Intervention strategies targeting mitophagy activation, restoration of mitochondrial fusion-fission homeostasis, mitochondrial antioxidant defense, and macrophage-targeted delivery have demonstrated potential for alleviating plaque burden and stabilizing vulnerable plaques in preclinical studies. This review systematically summarizes the role and molecular mechanisms of macrophage MQC imbalance in AS, outlines existing controversies, research gaps, and novel targeted intervention strategies, and provides a theoretical basis for the development of new therapeutic targets for AS prevention and treatment.

## 1 Introduction

Cardiovascular and cerebrovascular diseases are the leading causes of death worldwide. Current clinical management relies primarily on dietary interventions, exercise, and statin-based lipid-lowering agents. Although these strategies can reduce risk factors, significant residual cardiovascular risk remains, suggesting that lipid control alone is insufficient to halt the progression of atherosclerosis (AS) (Nevoit et al., 2025). AS is a chronic, low-grade inflammatory disease of the arterial wall, and sustained inflammatory amplification serves as the core driving force behind plaque formation, progression, and eventual rupture (Qu et al., 2022). Monocytes/macrophages are the predominant immune cells within plaques, maintaining chronic vascular inflammation through the secretion of inflammatory cytokines and chemokines and transforming into foam cells upon uptake of oxidized low-density lipoprotein, thereby directly driving the pathological process of AS (Xue et al., 2023). Therefore, elucidating the key molecular mechanisms by which macrophages drive inflammation is crucial for developing effective anti-inflammatory therapies with fewer side effects. Aging, an independent risk factor for AS, is closely associated with mitochondrial dysfunction, and failure of mitochondrial quality control (MQC) is both a trigger for cellular senescence and a critical node in the sustained amplification of the senescent phenotype (Salnikova et al., 2021). Mitochondria are not only responsible for energy metabolism but also regulate reactive oxygen species (ROS) production, innate immune activation, and macrophage polarization, with their dynamic balance being essential for immune homeostasis (Zhong et al., 2025). Recent studies have revealed that the MQC network (including mitophagy, fusion-fission balance, and mitochondrial DNA homeostasis) constitutes the core mechanism for maintaining normal mitochondrial structure and function (Ciccarelli et al., 2023; Zhong et al., 2025; Liu et al., 2026), and disruption of this system leads to the accumulation of damaged mitochondria, excessive ROS production, and inflammasome activation, consequently exacerbating macrophage inflammatory responses and foam cell formation. Cells carrying AS-susceptible mitochondrial mutations may exhibit fission abnormalities, which further implicate aberrant mitochondrial regulation in AS pathogenesis (Orekhov et al., 2025). Although the role of MQC imbalance in AS has garnered widespread attention, the mechanism through which macrophage MQC imbalance initiates inflammation, promotes foam cell formation, and drives plaque progression remains unclear. This review systematically summarizes the mechanisms, key molecular pathways, and potential targeted intervention strategies for macrophage MQC imbalance in AS, with the aim of providing a theoretical basis for elucidating the inflammatory mechanisms of AS and developing novel therapeutic targets for prevention and treatment.

## 2 Factors that trigger MQC imbalance

MQC imbalance arises principally from two sources: mitochondrial DNA (mtDNA) mutations and disruption of the mitochondrial respiratory chain (Liu et al., 2026). mtDNA mutations can impair mitochondrial function and weaken the cell’s ability to efficiently synthesize ATP, resulting in defects in the mitochondrial ETC and compromised energy production (Salnikova et al., 2021). In addition to spontaneous somatic mtDNA mutations, a range of additional factors can cause an imbalance in MQC. These include damage to both the nuclear and mitochondrial genomes; inherited mutations in either mtDNA or nuclear DNA can directly alter mitochondrial architecture or function (Orekhov et al., 2025).

### 2.1 Environmental factors

Exposure to toxins, environmental pollutants, and certain drugs can damage mitochondria and disrupt their normal function (Chen et al., 2026). When the balance between free radicals and the antioxidant defense network is disrupted, oxidative stress can contribute to mitochondrial dysfunction. Mitochondrial function declines naturally with age, energy production decreases, and oxidative damage accumulates (Camacho-Encina et al., 2024), which contribute to multiple aging-associated health problems. Metabolic conditions, such as diabetes, obesity, and metabolic syndrome, can also compromise mitochondrial health through pathways related to insulin resistance, chronic inflammation, and dysregulated lipid metabolism (Stepaniuk et al., 2024).

### 2.2 Nutritional deficiency

An insufficient intake of essential nutrients, vitamins, minerals, and antioxidants can impair mitochondrial function. Mitochondrial function depends on the availability of a broad range of nutrients, and deficiencies can disrupt both energy production and overall cellular health. Impaired mitochondrial biogenesis, the process that generates new mitochondria, can lead to an imbalance in MQC. Exercise, caloric restriction, and specific signaling pathways can influence mitochondrial health by regulating mitochondrial biogenesis (Xia et al., 2023).

### 2.3 Mitochondrial dynamics

An imbalance between mitochondrial fusion and fission, two processes that govern intracellular mitochondrial morphology and network architecture, is another important contributor to MQC imbalance (Ciccarelli et al., 2023). When these processes are dysregulated, mitochondrial function and cellular energy metabolism are affected. Mitochondrial mutations can give rise to a family of syndromes collectively termed mitochondrial diseases (cytopathies), which involve multiple organs and systems across the body (Li et al., 2022).

AS is a chronic inflammatory disease, and plaque regions have a hypoxic microenvironment. Within plaques, macrophages are the most abundant immune cell type. They convert into foam cells after engulfing oxLDL, a transformation that imposes heavy metabolic stress on mitochondria. The following sections describe the specific mitochondrial defects that arise in this setting.

## 3 Early-stage atherosclerosis: excessive lipid accumulation triggers defective mitochondrial fission and initiates chronic inflammation

The earliest phase of AS involves localized lipid deposition within the arterial intima, both inside cells and in the extracellular space. Circulating low-density lipoproteins (LDL) accumulate within the intima (Li et al., 2023). By the time lipid particles form in the intima, their chemical and physical properties diverge markedly from those of native LDL (Zhong et al., 2025). Macrophages and large perivascular cells are the dominant phagocytic cells within the intima. Smooth muscle cells can also internalize LDL and accumulate intracellular lipids, but to a far lesser degree than professional phagocytes. Lipid uptake proceeds hand in hand with cytokine release, and the released cytokines, in turn, promote further lipid deposition and recruit more immune cells to the inflammatory focus (Li et al., 2023). Lipid uptake stimulates the secretion of pro-inflammatory cytokines, which recruit additional immune cells to the site. Under normal conditions, the inflammatory response resolves as an anti-inflammatory response. However, when the innate inflammatory machinery is compromised, acute inflammation transitions to a chronic state, which fuels the continued evolution of atherosclerotic lesions across subsequent stages (Li et al., 2022).

In individuals whose innate immune function is intact, inflammation resolves rapidly. When the immune response is defective, rapid resolution fails, and the inflammatory reaction becomes entrenched as a chronic process (Orekhov et al., 2025). Previous studies indicated that the hypoxic microenvironment of AS can provoke mitochondrial mutations, and this is a key driver of inflammatory chronicity; cells carrying pro-atherosclerotic mitochondrial mutations show defective mitochondrial fission; they receive fission-stimulation signals but fail to execute mitochondrial fission normally (Orekhov et al., 2025). It is precisely these mitochondrially defective cells that display an impaired immune response, characterized by a pattern of “intolerant” cytokine secretion upon exposure to inflammatory stimuli. In a normal immune response, repeated inflammatory challenges cause cytokine output to progressively decline, the immune response gradually subsides, and inflammation ultimately resolves. However, in the intolerant state, repeated stimulation elicits cytokine secretion at levels comparable to or even exceeding those of the initial challenge (Xu et al., 2024). The net result is that the immune response fails to terminate, and inflammation continues to build and eventually develops into a chronic inflammatory response. Therefore, mitochondrial mutations are closely associated with the initiation and progression of atherosclerotic lesions. **Figure 1** summarizes these early events, contrasting the tolerant cytokine response of normal cells with the intolerant response of cells carrying pro-atherosclerotic mtDNA mutations and defective mitochondrial fission.

**Figure 1:**
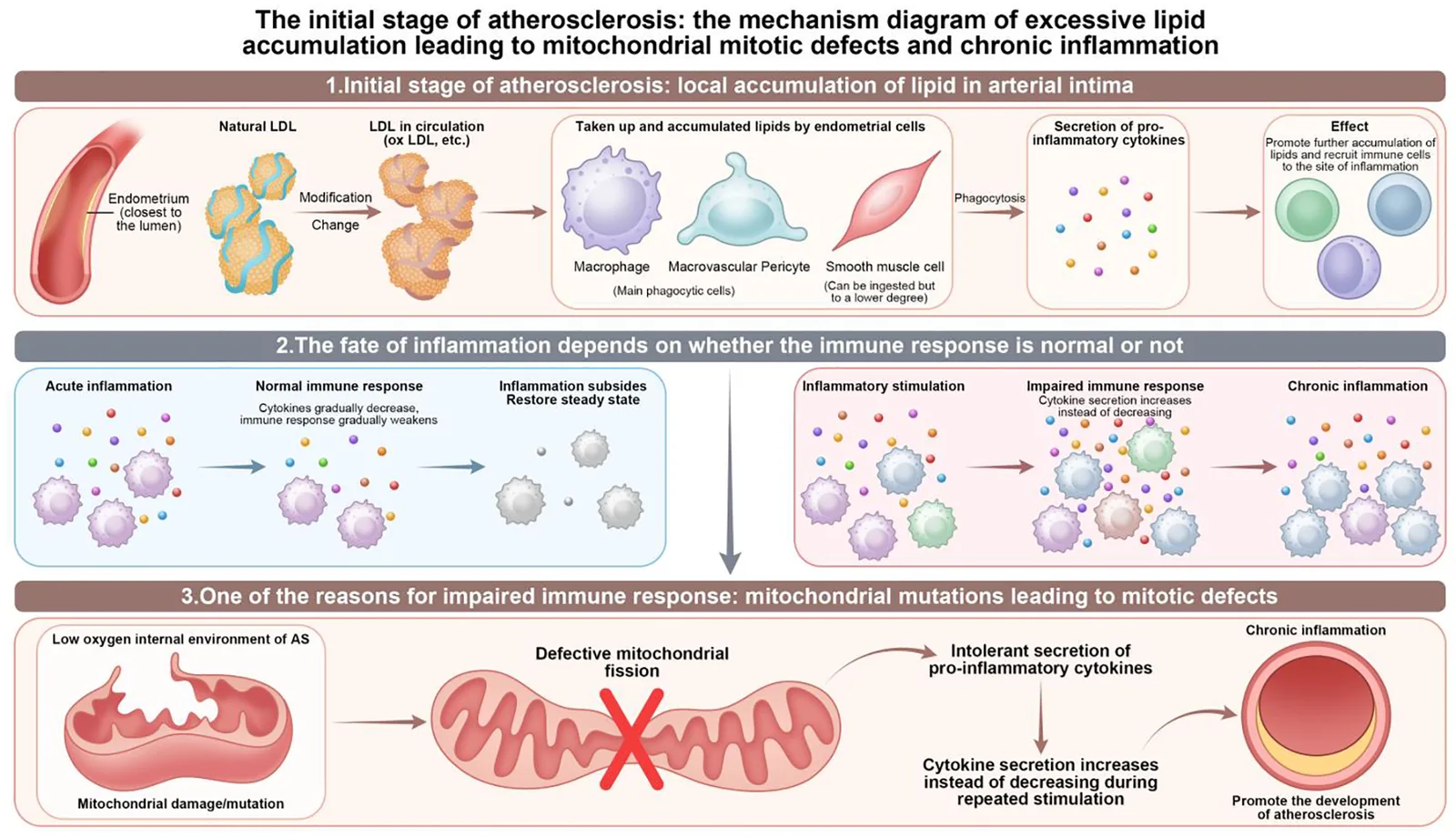
Defective mitochondrial fission links lipid accumulation to chronic inflammation in early atherosclerosis. In cells with normal (tolerant) immune responses, repeated inflammatory stimulation progressively reduces cytokine secretion, the immune response subsides, and inflammation resolves. In contrast, cells carrying pro-atherosclerotic mtDNA mutations exhibit defective mitochondrial fission and an “intolerant” phenotype, in which repeated stimulation maintains or even increases cytokine secretion; inflammation therefore fails to resolve and progresses to a chronic state that drives atherosclerotic lesion development.

## 4 Impaired macrophage mitophagy

Mitophagy is the principal mechanism underlying the clearance of damaged mitochondria. In advanced plaques, macrophage mitophagy is broadly impaired, allowing dysfunctional mitochondria to accumulate (Zhou et al., 2026). Under normal conditions, defective mitochondria are cleared through mitophagy, a specialized form of autophagy in which the organelle is enclosed within a double-membrane autophagosome that subsequently fuses with lysosomes to degrade its contents. Mitochondria play a vital role in innate immune signal transduction, and oxidative stress that damages mitochondrial function cooperates with defective mitochondrial fission to advance pathology (Stamerra et al., 2022). Mitophagy is a selective subtype of autophagy and is a core component of MQC (Zhou et al., 2026). As a self-limiting mechanism that maintains cellular homeostasis, mitophagy is an important regulatory pathway that controls excessive inflammation and preserves tissue homeostasis. When the mitophagic machinery induced by excessive pro-inflammatory cytokines is compromised, thereby blocking the lysosomal degradation of mitochondria, aging mitochondria begin to accumulate progressively (Xia et al., 2023).

The molecular chain linking impaired mitophagy to macrophage dysfunction is well defined. Loss of PINK1-Parkin function prevents the tagging and sequestration of depolarized mitochondria; defective organelles therefore accumulate, electron leakage rises, and mtDNA escapes into the cytosol, where it activates the cGAS-STING pathway and the NLRP3 inflammasome, driving IL-1β-dependent sterile inflammation and foam-cell formation (Zhou et al., 2026). Consistent with this framework, genetic loss of PINK1 or Parkin accelerates lesion development and destabilizes plaques in ApoE-/- mice, whereas restoring macrophage mitophagy limits lesion growth (Zhou et al., 2026); the direct manipulation evidence is consolidated in Section 6. **Figure 2** contrasts intact mitophagy, in which damaged mitochondria are packaged into autophagosomes and degraded by lysosomes, with the impaired mitophagy that allows damaged mitochondria to accumulate and sterile inflammation to become chronic.

**Figure 2:**
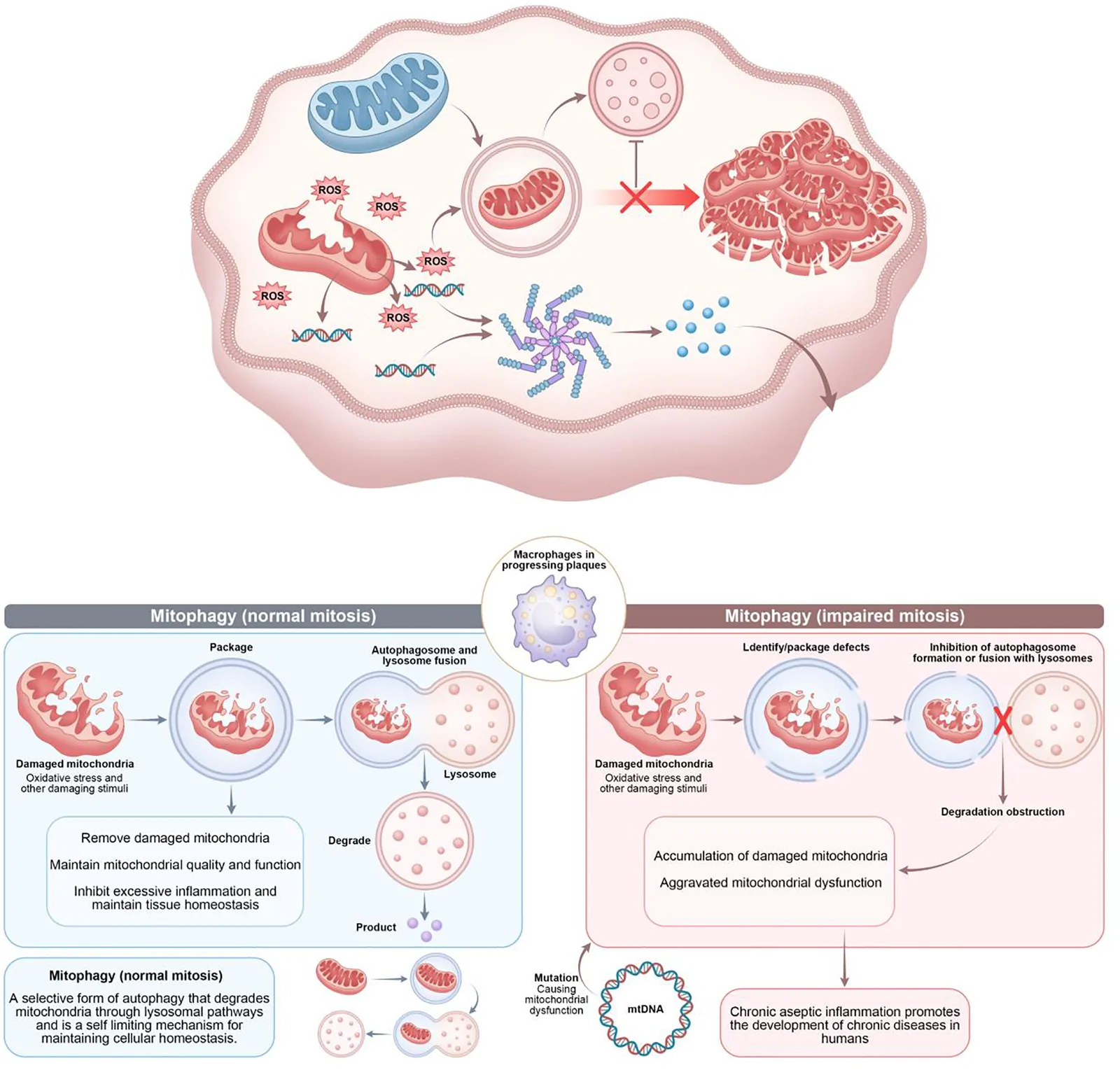
Progression from mitochondrial damage to its two alternative fates in plaque macrophages. Top, stepwise mitochondrial deterioration: mitochondria injured by the atherosclerotic environment (oxidative stress, damaged mtDNA, and respiratory-chain impairment) progressively lose membrane potential and function, and damaged organelles accumulate until the cell faces two alternative outcomes. Bottom, the two outcomes are compared side by side: Left, intact mitophagy: mitochondria damaged by oxidative stress are packaged into autophagosomes, which fuse with lysosomes so that their contents are degraded, maintaining mitochondrial quality and limiting inflammation. Right, impaired mitophagy: defective identification and packaging of damaged mitochondria, together with blocked autophagosome formation or lysosomal fusion, allow damaged mitochondria to accumulate; accumulating mtDNA mutations further aggravate mitochondrial dysfunction, and the resulting chronic sterile inflammation promotes human chronic diseases, including atherosclerosis.

## 5 Macrophage mitochondrial reprogramming drives atherosclerosis

Mitochondria orchestrate numerous metabolic processes within cells, including immune cells, and their core capability is large-scale ATP synthesis through oxidative phosphorylation (OXPHOS) (Jia et al., 2024). The principal enzymatic machinery is the ETC, which is embedded in the inner mitochondrial membrane and composed of five distinct protein complexes that sequentially transfer electrons from electron donors, typically products of nutrient oxidation in the tricarboxylic acid (TCA) cycle, to ultimately drive ATP synthase activity. OXPHOS is impaired as AS advances; the mtDNA copy number within foam cells decreases substantially, and the efficiency of mitochondrial energy production declines markedly (Chang et al., 2023).

Mitochondria are the central organelles for ROS generation and energy metabolism, and their shape changes through a continuous, dynamic process of outer and inner membrane fusion and fission. Recent studies have underscored the critical importance of mitochondrial dynamics (fusion and fission) in maintaining mitochondrial function, as these processes facilitate the distribution of metabolites, proteins, and DNA throughout the mitochondrial network (Chang et al., 2023). However, when fission becomes excessive, mitochondrial fragmentation ensues, ETC function is compromised, and mitochondrial ROS (mito-ROS) accumulates, ultimately damaging mammalian cells. During AS, an increase in ROS production occurs; the dysfunctional mitochondrial ETC can result in increased electron leakage, generating excessive ROS, which in turn damages mtDNA, proteins, and lipids, forming a self-reinforcing vicious cycle (Groh and Riksen, 2020; Batty et al., 2022).

During the progression of AS, mitochondrial reprogramming of macrophages is a key metabolic step that drives lesion progression. Under physiological conditions, macrophages rely mainly on OXPHOS to preserve lipid metabolic homeostasis and suppress inflammation (Xue et al., 2023). However, within the atherosclerotic microenvironment, sustained stimulation by pathogenic factors such as oxLDL triggers mitochondrial dynamic instability, TCA cycle disruption, and reduced activity of respiratory chain complexes. The metabolic mode consequently shifts from OXPHOS to glycolysis, a form of “aerobic glycolysis” known as the Warburg effect. This reprogramming not only reduces ATP synthesis efficiency but, more critically, provokes excessive mito-ROS production and the accumulation of metabolic intermediates (succinate, citrate, and others), which stabilize HIF-1α and activate the NLRP3 inflammasome, pushing macrophages toward a pro-inflammatory phenotype and accelerating both foam cell formation and lipid core expansion (Huang et al., 2024). Compounding the damage, cytosolic leakage of damaged mtDNA and impaired mitophagy further fuel the inflammatory cascade, weaken plaque stability, and raise the risk of rupture. Therefore, targeting mitochondrial metabolic reprogramming by restoring the fusion-fission balance, clearing dysfunctional mitochondria, or blocking key rate-limiting glycolytic enzymes holds promise as a novel therapeutic strategy for stabilizing atherosclerotic plaques (Su et al., 2023). **Figure 3** traces this cascade from oxLDL-driven MQC imbalance and the OXPHOS-to-glycolysis shift, through accumulation of succinate and citrate with HIF-1α stabilization and NLRP3 activation, to foam-cell formation and plaque instability, together with the corresponding intervention points.

**Figure 3:**
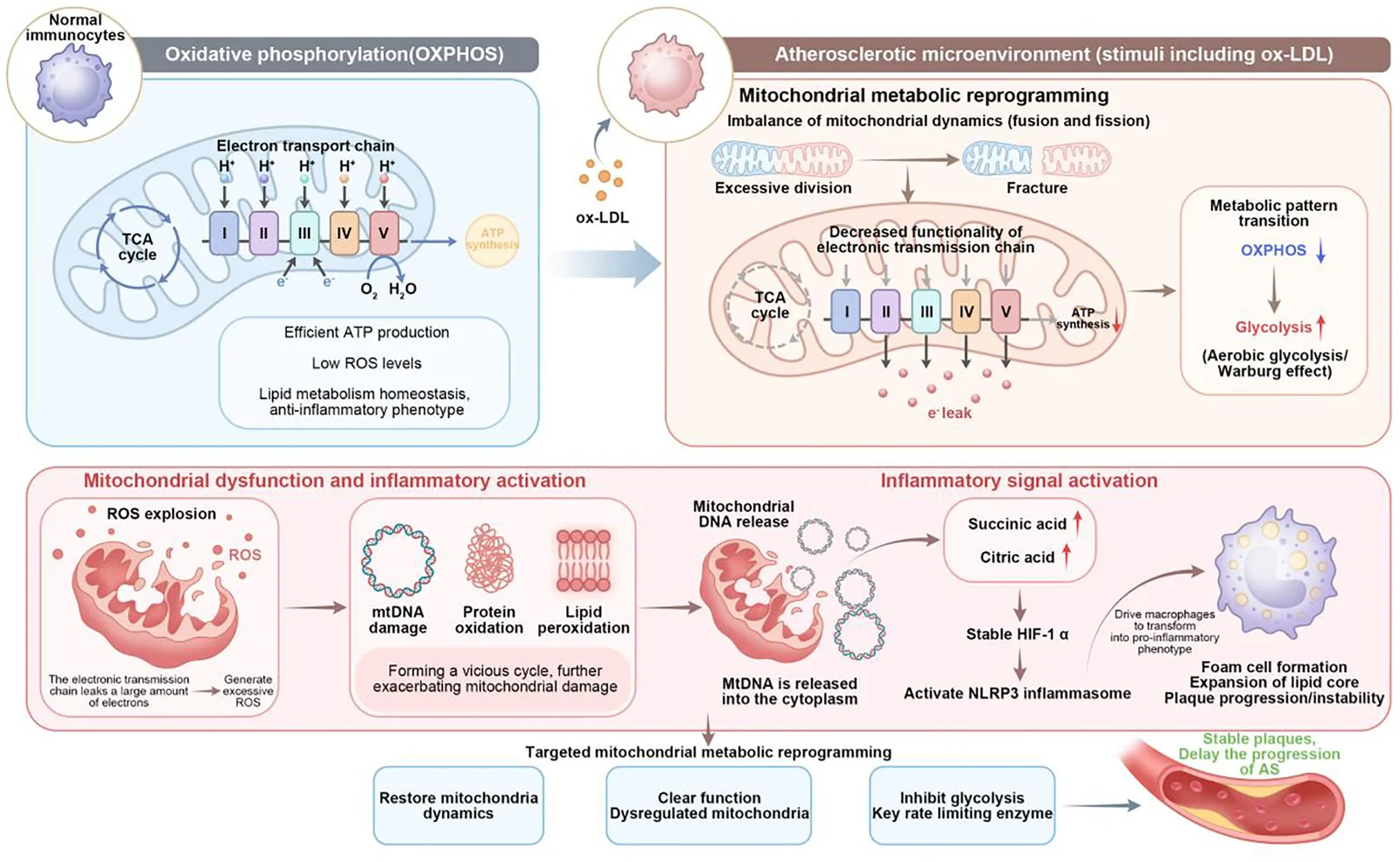
oxLDL-driven mitochondrial metabolic reprogramming in macrophages. Upper panels, in normal immune cells, intact oxidative phosphorylation (TCA cycle and electron transport chain complexes I-V) supports efficient ATP synthesis with limited ROS production; in the atherosclerotic microenvironment, sustained stimulation by oxLDL, hypoxia, and inflammatory factors triggers excessive fission, reduced respiratory-chain complex activity, and a metabolic shift from OXPHOS to aerobic glycolysis (the Warburg effect). Lower panels, electron leakage generates excessive ROS that damages mtDNA, proteins, and lipids in a self-reinforcing cycle, while succinate and citrate accumulate, stabilize HIF-1α, and activate the NLRP3 inflammasome, driving pro-inflammatory polarization, foam-cell formation, and plaque instability; the bottom row indicates the corresponding intervention points (restoring mitochondrial dynamics, clearing dysfunctional mitochondria, and inhibiting glycolytic rate-limiting enzymes).

## 6 Macrophage polarization and MQC imbalance are reciprocally coupled

Macrophage polarization has been reported to play a decisive role in the initiation and progression of AS (Zhong et al., 2025). Macrophages polarize into classically activated M1 phenotypes and alternatively activated M2 phenotypes. M1-polarized macrophages synthesize and release IL-6 and TNF-α, promoting plaque growth; M2-polarized macrophages exert protective effects by reducing plaque progression, and the anti-inflammatory factors they release (such as IL-10) contribute to their anti-inflammatory and protective functions. Therefore, the regulation of macrophage polarization is a new avenue for preventing and mitigating AS (Xue et al., 2023). Studies show that macrophages directly isolated from the plaques of patients with coronary artery disease display decreased mitochondrial membrane potential, elevated ROS generation, reduced ATP levels, and abnormal mitochondrial morphology (swelling and cristae disruption). In ApoE-/- mouse models of AS, genetic or pharmacological improvement of macrophage mitochondrial function by enhancing mitophagy or suppressing excessive fission significantly reduced plaque area and increased plaque stability, as reflected by a smaller necrotic core fraction (Su et al., 2023). When a macrophage MQC imbalance is experimentally induced in mice (by knocking out key mitophagy genes such as Parkin or PINK1), AS accelerates and more rupture-prone vulnerable plaques form (Zhou et al., 2026) (see Section 6).

During AS progression, macrophage polarization and MQC imbalance form a bidirectionally coupled relationship that jointly influences disease progression (Zakirov et al., 2020). Under physiological conditions, macrophages can exist in an M2-like anti-inflammatory state and depend on intact mitochondrial OXPHOS and fatty acid oxidation to sustain metabolic homeostasis and tissue repair. Under sustained exposure to oxLDL, the hypoxic plaque microenvironment, and inflammatory factors, imbalanced mitochondrial dynamics (enhanced fission and impaired fusion), reduced respiratory chain complex activity, and impaired mitophagy combine to produce MQC imbalance (Huang et al., 2024; Priya et al., 2024). Through metabolic reprogramming (a shift toward glycolysis) and accumulation of mito-ROS and metabolic intermediates, this dysfunction can stabilize HIF-1α and activate the NLRP3 inflammasome, promoting macrophage polarization toward the pro-inflammatory M1 phenotype and accelerating foam cell formation and necrotic core expansion. In turn, inflammatory factors (TNF-α, IL-1β, and others) secreted in the M1-polarized state further damage mitochondrial function, creating a self-sustaining positive feedback loop of “mitochondrial impairment → M1 polarization → inflammatory amplification → further mitochondrial damage” (Priya et al., 2024). Repairing mitochondrial function by promoting fusion or clearing damaged mitochondria can promote repolarization of macrophages toward the M2 phenotype, suggesting that intervention in the MQC system may be a potential strategy for breaking this vicious cycle and stabilizing atherosclerotic plaques (Wang et al., 2024; Zhou et al., 2026). **Figure 4** depicts this bidirectional coupling as two opposing routes: a vicious cycle in which MQC imbalance drives M1 polarization and M1-derived cytokines further damage mitochondria, and an intervention route in which restoring mitochondrial function promotes M2 repolarization and plaque stabilization.

**Figure 4:**
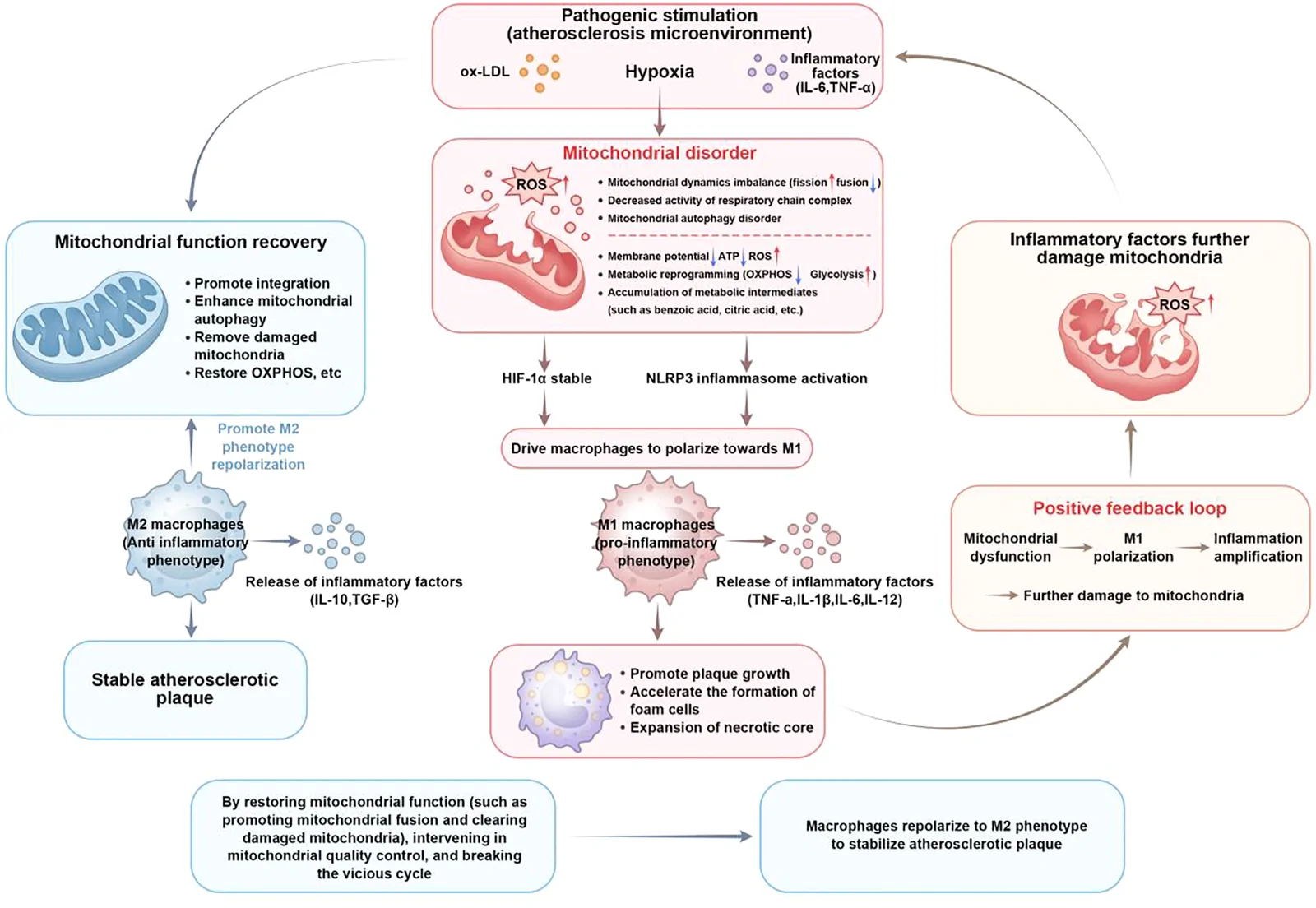
Reciprocal coupling between macrophage polarization and MQC imbalance. Pathogenic stimulation (oxLDL, hypoxia, IL-6, TNF-α) causes MQC imbalance (excessive fission, reduced respiratory-chain complex activity, disordered mitophagy), metabolic reprogramming toward glycolysis, accumulation of metabolic intermediates, HIF-1α stabilization, and NLRP3 activation, which together drive M1 polarization; M1-derived cytokines (TNF-α, IL-1β, IL-6, IL-12) in turn further damage mitochondria, forming a self-sustaining vicious cycle that accelerates foam-cell formation, necrotic-core expansion, and plaque growth. Conversely, restoring mitochondrial function (promoting fusion, enhancing mitophagy, clearing damaged mitochondria, restoring OXPHOS) favours M2 repolarization with IL-10 and TGF-β release and plaque stabilization.

Plaque macrophages are, however, highly heterogeneous, and the linear sequence of “glycolytic shift - M1 polarization - foam cell formation” should therefore be interpreted with caution. Single-cell transcriptomic studies of mouse and human atherosclerotic plaques have identified distinct macrophage subsets, including infiltrating inflammatory monocyte-derived macrophages, foamy macrophages with high expression of triggering receptor expressed on myeloid cells 2 (TREM2-high), and resident-like cells (Cochain et al., 2018; Depuydt et al., 2020). Importantly, non-foamy plaque macrophages, rather than foamy cells, are the predominant pro-inflammatory population in murine and human lesions, and TREM2-high foamy macrophages exhibit a distinct oxidative metabolic profile relative to inflammatory non-foamy cells (Cochain et al., 2018; Kim et al., 2018; Depuydt et al., 2020). These observations complicate the simple linear sequence proposed above. With respect to MQC, direct subset-resolved data remain scarce: MQC pathways (mitophagy, dynamics, biogenesis) have been manipulated almost exclusively in bulk macrophage populations, whereas MQC readouts for defined plaque subsets, such as TREM2-high foamy or resident-like macrophages, are largely lacking (Cochain et al., 2018; Kim et al., 2018; Depuydt et al., 2020). The MQC-inflammation axis described in this review should therefore be considered subset-dependent, and subset-resolved mitochondrial profiling of plaque macrophages represents a key research gap.

## 7 Direct experimental evidence: manipulating MQC pathways in macrophages alters atherosclerosis

The strongest support for a causal role of macrophage MQC in atherosclerosis comes from studies in which MQC pathways were experimentally manipulated and macrophage phenotypes and lesion outcomes were quantified. For mitophagy, genetic loss of the core mediators PINK1 and Parkin accelerates lesion development, increases inflammasome activation, and destabilizes plaques in ApoE-/- mice, establishing impaired mitophagy as a lesion-level mechanism rather than a bystander phenomenon (Zhou et al., 2026). For mitochondrial dynamics, Su et al. showed that the Drp1 inhibitor Mdivi-1 reduced plaque area, M1 polarization, NLRP3 activation, and DRP1 phosphorylation at Ser616 in high-fat-diet-fed ApoE-/- mice, and suppressed oxLDL-induced M1 polarization in THP-1-derived macrophages via the mito-ROS/NLRP3 axis (Su et al., 2023). Complementary mechanistic studies place Drp1-dependent fission upstream of macrophage inflammation: lipopolysaccharide induces inflammatory activation through Drp1-dependent fission (Kapetanovic et al., 2020), pro-inflammatory macrophage differentiation is associated with a Stat2-Drp1-mediated increase in mitochondrial mass (Yu et al., 2020), and, conversely, Drp1-mediated fission can protect macrophages from mtDNA/ZBP1-driven inflammation after myocardial infarction (Kondo et al., 2026), underscoring that the direction of the fission effect is context-dependent (see Section 10). The pharmacological strategies arising from these mechanisms are summarized in **Table 1**.

**Table 1:** Targeted intervention strategies against macrophage MQC imbalance in atherosclerosis.

Strategy/target	Representative agents	Mechanism of action	Evidence and model	Why translation has stalled
Mitophagy activation	Rapamycin, metformin, urolithin A	Enhanced clearance of damaged mitochondria via mTOR and AMPK-SIRT1 pathways (Zhou et al., 2026)	Preclinical (ApoE-/- mice, macrophage cell models)	Animal data only; optimal dosing and long-term safety of autophagy modulation in humans unresolved
Restoration of mitochondrial dynamics and membrane integrity	Mdivi-1 (Drp1 inhibitor), SS-31 (elamipretide)	Mdivi-1 blocks Drp1-dependent fission; SS-31 stabilizes cardiolipin and the inner mitochondrial membrane; both reduce mito-ROS, NLRP3 activation, and M1 polarization	Preclinical (high-fat-diet ApoE-/- mice)	Drp1 also has protective, context-dependent roles; off-target actions of Mdivi-1; no trials in patients
Mitochondria-targeted antioxidants	MitoQ, Mito-TEMPO	Direct scavenging of mitochondrial ROS; attenuates mtDNA damage	Preclinical in atherosclerosis; MitoQ improved endothelial function in a small randomized human trial	Antioxidant trials in cardiovascular disease largely negative; net benefit uncertain
NLRP3/IL-1β axis blockade	MCC950, colchicine, canakinumab	Inhibits inflammasome activation or neutralizes IL-1β downstream of MQC failure (Ridker et al., 2017; Penna and Pagliaro, 2025)	Clinical (CANTOS; colchicine trials)	Modest residual-risk reduction; infection risk and cost; upstream MQC defect not corrected
Mitochondrial H2S delivery	AP39	Improves mitochondrial bioenergetics; shifts the M1/M2 balance; reduces plaque macrophage content	Preclinical (ApoE-/- mice) (Stachowicz et al., 2024)	Compound stability and *in vivo* dosing unresolved; species differences; no clinical data
Inhibition of glycolytic reprogramming	Glycolysis inhibitors	Restores OXPHOS; promotes M2 repolarization	Preclinical	Systemic toxicity; macrophage selectivity not achieved
Macrophage-targeted nanodelivery	HDL-based and other nanoparticles	Selective delivery of mitoprotective cargo to plaque macrophages	Preclinical	Biodistribution, cargo stability, and scale-up challenges
mtDNA modification/editing	Pemetrexed (METTL4 inhibitor); mitochondria-targeted editors	Reduces mtDNA 6mA and mtDNA release; removes pro-atherogenic mutations in cybrid cells (Orekhov et al., 2025)	*In vitro* (cybrids)	*In vivo* delivery of editors to mitochondria unsolved

For the mitochondrial genome, direct evidence comes from cybrid experiments: hybrid cells carrying mitochondria from patients with atherosclerosis show defective mitophagy and intolerant inflammatory responses, and elimination of the single pro-atherosclerotic mutation m.15059G>A by mitochondrial genome editing restored both normal mitophagy and tolerant cytokine responses (Orekhov et al., 2025), a single-mutation-level demonstration of causality. Taken together, these manipulation studies indicate that MQC pathways in macrophages are functional determinants of plaque burden and composition rather than mere correlates. Nevertheless, macrophage-specific genetic evidence remains limited: most studies used global knockout or systemic pharmacological approaches, and macrophage-conditional manipulations with quantitative readouts of plaque composition (necrotic-core size, collagen content, lesional macrophage subsets) are still needed.

## 8 Mitochondrial genome mutations in atherosclerosis

Reports have linked specific mutations in the mitochondrial genome to cancer, whereas AS-related mitochondrial mutations display maternal inheritance patterns associated with susceptibility to AS. These data strongly implicate mitochondrial mutations in the development of atherosclerotic lesions (Orekhov et al., 2025). Although mtDNA has a relatively small molecular size, mutations in mtDNA may play significant roles in disease pathogenesis. Deeper functional characterization may establish mitochondrial mutations as promising diagnostic markers and identify mitochondrial mutation-associated diseases as tractable pharmacological targets (Orekhov et al., 2025).

A substantial body of research has confirmed the accumulation of mtDNA variants in elderly individuals, indicating that mtDNA mutations play a role in aging. In recent years, using hybrid cell lines carrying different mitochondrial genome variants isolated from patients with AS, investigators have confirmed the association between mitochondrial function and AS-related mitochondrial mutations (Wang et al., 2025). Earlier work extracting mtDNA from the leukocytes of patients with AS revealed correlations between several mtDNA mutations and AS. AS-associated mtDNA mutations can be homoplasmic (the mutation is present in all copies of mtDNA) or heteroplasmic (the mutation is present in a proportion of mtDNA copies) (Orekhov et al., 2025). New genetic technologies now provide tools for deeper exploration of the mitochondrial genome and have uncovered numerous mtDNA variants linked to human diseases (Liu et al., 2025).

Genetic variations in mtDNA may contribute to chronic conditions such as AS, hypertension, type 2 diabetes, and obesity. However, the mitochondrial genome is highly unstable (Wu et al., 2025). Studies have found that mitochondrial mutations can co-localize with different stages of atherosclerotic lesions and with gene loci in the human aortic intima, and that a positive correlation exists between the number of mutated genes and the severity of atherosclerotic lesions (Orekhov et al., 2025). The higher the mutation burden in the mitochondrial genome, the more prominent the corresponding lesion, and in regions with the most advanced pathology, the mutation burden was also highest.

mtDNA mutations may be linked to the degree of monocyte activation: in monocytes obtained from asymptomatic patients with AS, a correlation exists between the extent of pro-inflammatory activation and the occurrence of AS-associated mtDNA mutations, suggesting that certain mutations may alter the activation program of monocyte-derived macrophages in AS (Orekhov et al., 2025). In an atherosclerotic setting, the mitochondrial genome of macrophages is not merely a collection of energy metabolism-related genes; its dysfunction and epigenetic modifications have become important drivers of inflammation and disease progression. Activation of vascular cell adhesion molecule-1 (VCAM-1) in the inflammatory environment can induce abnormal mtDNA synthesis and leakage into the cytoplasm, amplifying inflammatory responses through the STING pathway and establishing a positive feedback loop (Natarajan et al., 2024). In contrast, elevated levels of METTL4 in macrophages lead to the N6-methyladenine (6mA) modification of mtDNA, which suppresses MT-ATP6 gene expression, disrupts respiratory chain function, and drives mtDNA release. This leaked mtDNA functions as a damage-associated molecular pattern (DAMP), activating immune pathways, including cGAS-STING, the NLRP3 inflammasome, and TLR9, exacerbating oxidative stress and inflammation while simultaneously pushing macrophages toward M1 pro-inflammatory polarization and accelerating plaque formation and instability (Zheng et al., 2025). The METTL4 inhibitor pemetrexed has been identified as a compound that can target these processes and has been shown to delay AS progression in animal models, indicating that mtDNA modifications and their related pathways are promising therapeutic targets (Zheng et al., 2025).

In addition, mitochondrial mutations in AS can alter mitochondrial dynamics, promoting fusion (to cope with stress) and fission (to remove damaged portions). When fission becomes excessive and fusion falls behind, large numbers of fragmented, functionally compromised mitochondria are produced, ultimately leading to a dynamic imbalance (Li et al., 2021). **Figure 5** integrates these mtDNA-centred mechanisms: mutation load rises with lesion stage, METTL4-mediated 6mA modification of mtDNA suppresses MT-ATP6 and promotes mtDNA release, and the released mtDNA activates cGAS-STING, the NLRP3 inflammasome, and TLR9, driving M1 polarization, foam-cell formation, and plaque instability, whereas METTL4 inhibition with pemetrexed stabilizes plaques in experimental models.

**Figure 5:**
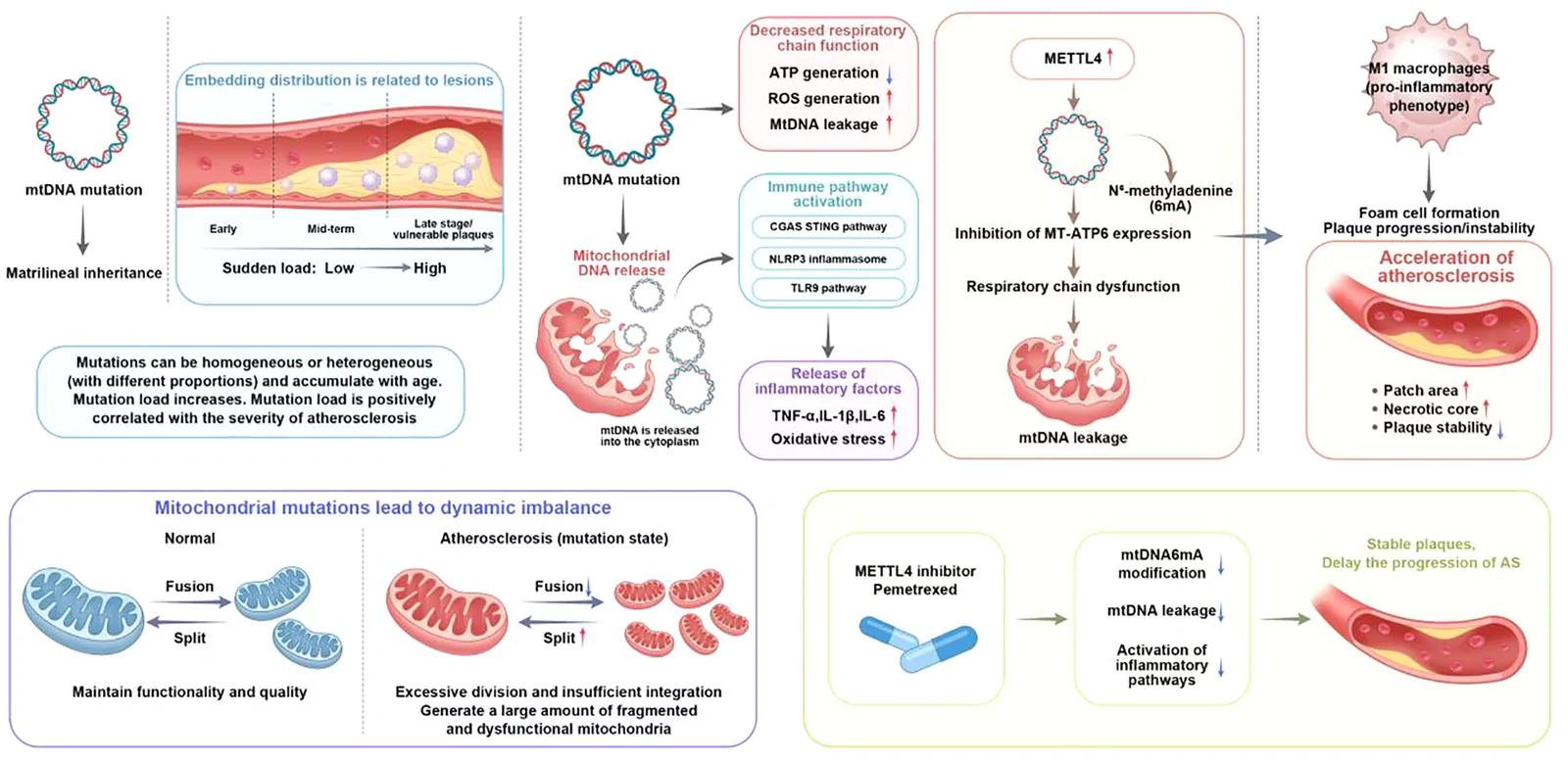
mtDNA mutations and epigenetic modifications as drivers of macrophage inflammation in atherosclerosis. Left, atherosclerosis-associated mtDNA mutations show maternal (matrilineal) inheritance, and their burden correlates positively with lesion severity from early lesions to late vulnerable plaques. Centre, mtDNA mutations impair respiratory-chain function (reduced ATP, increased ROS) and promote mtDNA release into the cytoplasm, where released mtDNA activates the cGAS-STING, NLRP3-inflammasome, and TLR9 pathways and drives the release of TNF-α, IL-1β, and IL-6. Right, elevated METTL4 increases N6-methyladenine (6mA) modification of mtDNA, suppresses MT-ATP6 expression, and further aggravates respiratory-chain dysfunction and mtDNA leakage, driving M1 polarization, foam-cell formation, and plaque instability; the METTL4 inhibitor pemetrexed reduces 6mA modification and mtDNA release and stabilizes plaques in experimental models.

## 9 Distinguishing macrophage MQC imbalance from MQC imbalance in other cell types in atherosclerosis

In the pathological context of AS, MQC imbalance in macrophages differs fundamentally from that in other cell types (VSMCs and endothelial cells) in both mechanistic terms and pathological significance (Atici et al., 2023).

First, their functional consequences are distinct. The core consequence of macrophage MQC imbalance is metabolic reprogramming (aerobic glycolysis), which drives M1 inflammatory polarization and foam cell formation, directly expanding the lipid core and weakening plaque stability. MQC imbalance in VSMCs commonly leads to phenotypic switching (from contractile to synthetic), excessive proliferation, and aberrant extracellular matrix remodeling, contributing to fibrous cap thinning. MQC imbalance in endothelial cells drives elevated oxidative stress, increased permeability, and upregulation of adhesion molecules, thereby mediating the initiation of endothelial injury and monocyte recruitment (Qu et al., 2022; Singh et al., 2026).

Second, as the most abundant immune cells in plaques, macrophages occupy a central position in the inflammatory cascade. Through NLRP3 inflammasome activation and IL-1β/IL-18 secretion, macrophage MQC imbalance can amplify and perpetuate microenvironmental injury throughout the plaque, affecting the functional status of neighboring endothelial cells and smooth muscle cells. In contrast, MQC imbalance in smooth muscle and endothelial cells manifests as localized structural damage and a decline in barrier function (Huynh and Heo, 2021; Ren et al., 2024).

Third, the target specificities of the intervention strategies differ. Interventions directed at macrophage MQC imbalance, such as restoring mitophagy or inhibiting key glycolytic enzymes, can not only reverse their own pro-inflammatory phenotype but also indirectly optimize the overall metabolic homeostasis of the vessel wall. Therapeutic strategies targeting mitochondria in other cell types must simultaneously accommodate their unique synthetic and barrier functions (Atici et al., 2023).

In summary, based on the currently available evidence—obtained predominantly from bulk macrophage studies in murine models—macrophage MQC imbalance appears to play a central dual role in AS as both an inflammatory hub and a metabolic switch, thereby influencing inflammatory responses and plaque instability (Zhong et al., 2025). MQC imbalance in other cells (smooth muscle and endothelial cells) is generally considered to act as a synergistic or downstream effector affecting plaque structural integrity and vascular barrier function. We emphasize that this hierarchy among cell types is a working model rather than an experimentally established conclusion: direct, side-by-side manipulation of MQC in macrophages, VSMCs, and endothelial cells within the same *in vivo* atherosclerosis model has not been reported, and such comparative studies represent an important research gap. Therefore, targeting macrophage mitochondrial reprogramming may simultaneously achieve anti-inflammatory, metabolic remodeling, and immunomodulatory benefits, representing an intervention strategy with substantial translational potential (Liu et al., 2026).

## 10 Clinical and therapeutic significance: therapeutic targets

Regarding MQC imbalance in macrophages, preclinical studies have developed various intervention strategies targeting mitophagy, fusion-fission homeostasis, oxidative stress, and inflammatory pathways. Targeted activation of mitophagy promotes the clearance of damaged mitochondria. Rapamycin, metformin, and urolithin A enhance macrophage mitophagy by regulating the mTOR and AMPK-SIRT1 pathways, thereby reducing ROS levels, inhibiting foam cell formation, and attenuating plaque inflammation. To address excessive mitochondrial fission, the Drp1 inhibitor Mdivi-1 and the mitochondrial protective peptide SS-31 can ameliorate the fusion-fission imbalance and preserve mitochondrial network integrity. Mitochondria-targeted antioxidants such as MitoQ and Mito-TEMPO directly scavenge mitochondrial ROS, alleviate mtDNA damage, and mitigate oxidative stress in macrophages. Downstream, MCC950 and colchicine can be employed to inhibit NLRP3 inflammasome activation, reducing the release of pro-inflammatory cytokines such as IL-1β and IL-18. In addition, inhibiting macrophage glycolysis and promoting oxidative phosphorylation can reprogram macrophages toward an anti-inflammatory M2 phenotype. Macrophage-targeted nanodelivery systems enable targeted delivery of drugs to plaque sites, reducing systemic adverse effects. Although these approaches have demonstrated anti-atherosclerotic potential in animal models, most remain confined to preclinical research, and their translation into clinical practice still faces numerous challenges. **Table 1** summarizes these strategies, together with the level of evidence supporting each and the reasons why clinical translation has stalled so far.

## 11 Existing controversies and research gaps

Several controversies and open questions temper the framework developed above. First, cause versus consequence: it remains unclear whether mitochondrial and metabolic abnormalities in plaque macrophages initiate lesion development or arise secondarily to the plaque microenvironment, because most available data are cross-sectional (You et al., 2025). Second, macrophage heterogeneity challenges the linear “glycolytic shift - M1 polarization - foam cell formation” sequence: single-cell analyses indicate that the most inflammatory plaque macrophages are generally non-foamy, whereas TREM2-high foamy macrophages are metabolically and functionally distinct (Cochain et al., 2018; Kim et al., 2018; Depuydt et al., 2020). Third, the role of mitochondrial fission appears bidirectional and context-dependent: whereas inhibition of Drp1-dependent fission attenuates NLRP3 activation and M1 polarization in atherosclerosis models, Drp1-mediated fission has also been shown to protect macrophages from mtDNA/ZBP1-driven inflammation in post-infarct cardiac remodelling (Kondo et al., 2026). Fourth, the evidence base is dominated by RAW264.7 cells and ApoE-/- mice, which do not faithfully reproduce human disease, and subset-resolved MQC measurements in human plaques are almost entirely missing. Resolving these controversies requires longitudinal intervention studies establishing causality, subset-specific MQC profiling, and head-to-head comparison of MQC manipulation across macrophages, VSMCs, and endothelial cells within the same disease model.

## 12 Summary and conclusions

Macrophages stand at the front line of maladaptive immune cell activation and infiltration, while simultaneously serving as the principal hub for intercellular communication, stimulating immune cells and tissue-resident cells to produce broad pathological effects in the vessel wall. Elucidating the molecular mechanisms underlying this shift toward a pro-inflammatory, infiltrative phenotype constitutes a potential therapeutic entry point for vascular diseases (Zhong et al., 2025). A large body of evidence indicates that mitochondrial and metabolic abnormalities may be fundamental mechanisms driving macrophage activation and pathological activity. However, more work is needed to identify the precise spatiotemporal factors and biochemical determinants underlying these processes and to clearly establish whether mitochondrial and metabolic abnormalities are the causes or consequences of vascular disease (You et al., 2025).

This situation differs when subendothelial cells carry proatherosclerotic mutations in mtDNA. Such mutations are associated with defective mitochondrial fission, and defective mitochondrial fission in turn disrupts the innate immune response characterized by intolerant cytokine secretion (Orekhov et al., 2025). These cells continuously secrete far larger quantities of pro-inflammatory cytokines than do cells without proatherosclerotic mutations, drawing more immune cells into the inflammatory region. Some recruited cells may carry proatherosclerotic mtDNA mutations, which further exacerbate the chronicity of inflammation (Orekhov et al., 2025). Chronic local inflammation of the arterial wall can persist for years, eventually manifesting as macroscopic atherosclerotic plaques that threaten the patient’s health and life. Although mtDNA is small, its role in pathogenesis should not be underestimated. Further exploration may establish mitochondrial mutations as valuable diagnostic markers and identify mutation-driven diseases as pharmacological targets (Orekhov et al., 2025).

Macrophages have opened an exciting avenue for medical research on multiple vascular diseases. Targeting macrophage mitochondrial dynamics as a therapeutic entry point for vascular diseases is a potential therapeutic strategy (Su et al., 2023). Zakirov et al. (2020) have systematically reviewed gene therapies targeting macrophage mitochondria in the atherosclerotic context. The complexity of signaling pathways, the diversity of macrophage paracrine effects during vascular disease progression, and the inherent limitations of cell culture and mouse models in faithfully reproducing human disease complexity have hindered the development of direct mitochondria-targeted therapies. The distinct pathophysiological features of different diseases pose unique therapeutic challenges, and the widespread distribution of plaques in AS makes it particularly difficult to target any single plaque in isolation (Liu et al., 2026). Existing therapeutic agents aimed at intervening in macrophage metabolic alterations have significant off-target effects. To reduce adverse systemic consequences, future research should focus on identifying macrophage-specific therapeutic targets. Therefore, applying transcriptomic approaches to explore the mitochondrial and metabolic basis of macrophages *in vivo* is especially critical (Cochain et al., 2018; Kim et al., 2018). Most studies cited in this review used RAW264.7 cells or ApoE-/- mouse models. Because these models have inherent limitations, their results may not fully reflect the role of mitochondrial fission in humans. Future studies should incorporate other macrophage cell lines, such as THP-1, to comprehensively dissect the regulatory mechanisms of mitochondrial fission in M1 polarization-associated AS.

Mitochondria are highly dynamic organelles that continuously undergo fusion (two or more mitochondria merge into one) and fission (one mitochondrion divides into two). Balancing these processes is indispensable for maintaining cellular homeostasis (Nevoit et al., 2025). In cardiovascular and other diseases, the balance tilts toward increased fission and reduced fusion, with an overall increase in mitochondrial fragmentation (Poznyak et al., 2022). This process typically triggers profound remodeling of cellular and metabolic activities, as mitochondrial fragmentation is closely linked to elevated mitochondrial superoxide levels, increased intracellular ROS, enhanced apoptosis, cellular phenotypic transdifferentiation, and metabolic reprogramming, characterized by enhanced glycolysis and diminished OXPHOS (Pozo Giráldez et al., 2026). The metabolic shift from OXPHOS to glycolysis, known as the Warburg effect, has been documented in many diseases and cell types, including vascular diseases. However, in the context of vascular disease, it remains unclear whether these mitochondrial and metabolic changes precede disease onset (and thus represent key initiating mechanisms) or are merely downstream epiphenomena triggered by disease pathology (You et al., 2025).

During vascular disease progression, macrophages have been shown to possess altered mitochondrial and metabolic characteristics that align with macrophage mobilization, phenotypic switching, and accumulation (Zhang et al., 2026). This review summarizes studies of mitochondrial and metabolic abnormalities in macrophages during vascular diseases (Zhong et al., 2025). Cell-specific therapeutic delivery models may represent one route to overcome the current impasse, improving the therapeutic targeting of macrophages while minimizing off-target effects. One particularly exciting direction is the use of nanoparticle technologies (Xu et al., 2025). Nanoparticle technology can improve the bioavailability and stability of encapsulated therapeutic agents. Therapeutic cargoes can span multiple categories, including proteins, drugs, peptides, siRNAs, and miRNAs. This technology encapsulates bioactive cargo within nanoparticles, which are typically composed of lipids. In the field of vascular diseases, this technology may enable the delivery of macrophage-specific therapeutic interventions. Although nanoparticles still have limitations, the preclinical data obtained using this technology have shown considerable promise (Zakirov et al., 2020). Integrating nanoparticle technology with recent advances in transcriptomics and proteomics will provide valuable tools for elucidating the *in vivo* roles of macrophages during the progression of multiple diseases, which has previously been extremely difficult. This makes it possible to simultaneously target one or more specific macrophage phenotypes, thereby minimizing the off-target effects of macrophage-targeted therapy while maximizing the delivery efficiency of therapeutic agents to pathogenic macrophage subpopulations. Overall, cell-specific therapeutic delivery models directed against macrophage subpopulations offer a promising therapeutic avenue for combating vascular diseases (Liu et al., 2026).

## References

[B1] Atici A. E. Crother T. R. Noval Rivas M. (2023). Mitochondrial quality control in health and cardiovascular diseases. Front. Cell Dev. Biol. 11, 1290046. doi: 10.3389/fcell.2023.1290046 38020895 PMC10657886

[B2] Batty M. Bennett M. R. Yu E. (2022). The role of oxidative stress in atherosclerosis. Cells 11, 3843. doi: 10.3390/cells11233843 36497101 PMC9735601

[B3] Camacho-Encina M. Booth L. K. Redgrave R. E. Folaranmi O. Spyridopoulos I. Richardson G. D. (2024). Cellular senescence, mitochondrial dysfunction, and their link to cardiovascular disease. Cells 13, 353. doi: 10.3390/cells13040353 38391966 PMC10886919

[B4] Chang X. Liu R. Li R. Peng Y. Zhu P. Zhou H. (2023). Molecular mechanisms of mitochondrial quality control in ischemic cardiomyopathy. Int. J. Biol. Sci. 19, 426–448. doi: 10.7150/ijbs.76223 36632466 PMC9830521

[B5] Chen T. H. Chu I. T. Chang R. Y. Wang H. C. Chung C. J. Tsai T. H. . (2026). Microplastics induce mitochondrial dysfunction and accelerate cardiovascular pathogenesis. Arch. Toxicol. 100, 1699–1711. doi: 10.1007/s00204-026-04327-w 41762287 PMC13086901

[B6] Ciccarelli G. Conte S. Cimmino G. Maiorano P. Morrione A. Giordano A. (2023). Mitochondrial dysfunction: The hidden player in the pathogenesis of atherosclerosis? Int. J. Mol. Sci. 24, 1086. doi: 10.3390/ijms24021086 36674602 PMC9861427

[B7] Cochain C. Vafadarnejad E. Arampatzi P. Pelisek J. Winkels H. Ley K. . (2018). Single-cell RNA-seq reveals the transcriptional landscape and heterogeneity of aortic macrophages in murine atherosclerosis. Circ. Res. 122, 1661–1674. doi: 10.1161/CIRCRESAHA.117.312509 29545365

[B8] Depuydt M. A. C. Prange K. H. M. Slenders L. Örd T. Elbersen D. Boltjes A. . (2020). Microanatomy of the human atherosclerotic plaque by single-cell transcriptomics. Circ. Res. 127, 1437–1455. doi: 10.1161/CIRCRESAHA.120.316770 32981416 PMC7641189

[B9] Groh L. A. Riksen N. P. (2020). Macrophage mitochondrial superoxides as a target for atherosclerotic disease treatment. Int. J. Biochem. Cell Biol. 129, 105883. doi: 10.1016/j.biocel.2020.105883 33176186

[B10] Huang H. Li G. He Y. Chen J. Yan J. Zhang Q. . (2024). Cellular succinate metabolism and signaling in inflammation: Implications for therapeutic intervention. Front. Immunol. 15, 1404441. doi: 10.3389/fimmu.2024.1404441 38933270 PMC11200920

[B11] Huynh D. T. N. Heo K. S. (2021). Role of mitochondrial dynamics and mitophagy of vascular smooth muscle cell proliferation and migration in progression of atherosclerosis. Arch. Pharm. Res. 44, 1051–1061. doi: 10.1007/s12272-021-01360-4 34743301

[B12] Jia G. Bai H. Mather B. Hill M. A. Jia G. Sowers J. R. (2024). Diabetic vasculopathy: Molecular mechanisms and clinical insights. Int. J. Mol. Sci. 25, 804. doi: 10.3390/ijms25020804 38255878 PMC10815704

[B13] Kapetanovic R. Afroz S. F. Ramnath D. Lawrence G. M. Okada T. Curson J. E. . (2020). Lipopolysaccharide promotes Drp1-dependent mitochondrial fission and associated inflammatory responses in macrophages. Immunol. Cell Biol. 98, 528–539. doi: 10.1111/imcb.12363 32686869 PMC7497224

[B14] Kim K. Shim D. Lee J. S. Zaitsev K. Williams J. W. Kim K. W. . (2018). Transcriptome analysis reveals nonfoamy rather than foamy plaque macrophages are proinflammatory in atherosclerotic murine models. Circ. Res. 123, 1127–1142. doi: 10.1161/CIRCRESAHA.118.312804 30359200 PMC6945121

[B15] Kondo Y. Koga J. I. Orkhonselenge N. Wang L. Hasuzawa N. Katsuki S. . (2026). Drp1-mediated mitochondrial fission protects macrophages from mtDNA/ZBP1-mediated inflammation and inhibits post-infarct cardiac remodelling. Cardiovasc. Res. 122, 214–229. doi: 10.1093/cvr/cvag006 41544243

[B16] Li Y. Liu Y. Liu S. Gao M. Wang W. Chen K. . (2023). Diabetic vascular diseases: molecular mechanisms and therapeutic strategies. Signal. Transduct. Target. Ther. 8, 152. doi: 10.1038/s41392-023-01400-z 37037849 PMC10086073

[B17] Li Y. Ma Y. Dang Q. Y. Fan X. R. Han C. T. Xu S. Z. . (2022). Assessment of mitochondrial dysfunction and implications in cardiovascular disorders. Life Sci. 306, 120834. doi: 10.1016/j.lfs.2022.120834 35902031

[B18] Li D. Yang S. Xing Y. Pan L. Zhao R. Zhao Y. . (2021). Novel insights and current evidence for mechanisms of atherosclerosis: Mitochondrial dynamics as a potential therapeutic target. Front. Cell Dev. Biol. 9, 673839. doi: 10.3389/fcell.2021.673839 34307357 PMC8293691

[B19] Liu X. Wang C. Guan X. (2025). Mitochondrial dysfunction is a potential key mechanism for atherosclerosis predisposition in patients with systemic lupus erythematosus. J. Autoimmun. 157, 103488. doi: 10.1016/j.jaut.2025.103488 40966991

[B20] Liu Y. Luo N. Xi X. Hou J. Li X. Xia M. . (2026). Mitochondrial dysfunction and applications of mitochondrial-targeted delivery systems in atherosclerosis. Drug Delivery 33, 2627689. doi: 10.1080/10717544.2026.2627689 41668545 PMC12895878

[B21] Natarajan N. Florentin J. Johny E. Xiao H. O'Neil S. P. Lei L. . (2024). Aberrant mitochondrial DNA synthesis in macrophages exacerbates inflammation and atherosclerosis. Nat. Commun. 15, 7337. doi: 10.1038/s41467-024-51780-1 39187565 PMC11347661

[B22] Nevoit G. Jarusevicius G. Potyazhenko M. Mintser O. Bumblyte I. A. Vainoras A. (2025). Mitochondrial dysfunction and atherosclerosis: The problem and the search for its solution. Biomedicines 13, 963. doi: 10.3390/biomedicines13040963 40299559 PMC12024619

[B23] Orekhov A. N. Orekhov N. A. Sukhorukov V. N. Khotina V. A. Kovianova T. I. Sobenin I. A. (2025). Mitochondrial DNA mutations as a factor in the heritability of atherosclerosis and other diseases. Curr. Med. Chem. 32, 6929–6940. doi: 10.2174/0109298673291199241129044139 39817381 PMC12645116

[B24] Penna C. Pagliaro P. (2025). Endothelial dysfunction: Redox imbalance, NLRP3 inflammasome, and inflammatory responses in cardiovascular diseases. Antioxid. (Basel). 14, 256. doi: 10.3390/antiox14030256 40227195 PMC11939635

[B25] Poznyak A. V. Kirichenko T. V. Borisov E. E. Shakhpazyan N. K. Kartuesov A. G. Orekhov A. N. (2022). Mitochondrial implications in cardiovascular aging and diseases: The specific role of mitochondrial dynamics and shifts. Int. J. Mol. Sci. 23, 2951. doi: 10.3390/ijms23062951 35328371 PMC8949229

[B26] Pozo Giráldez A. Bravo Gómez A. Calmarza P. Sienes Bailo P. Dayaldasani Khialani A. Montolio Breva S. . (2026). Oxidative stress and its role in vascular damage and atherosclerosis. Int. J. Mol. Sci. 27, 1075. doi: 10.3390/ijms27021075 41596717 PMC12841722

[B27] Priya H. K. Jha K. P. Kumar N. Singh S. (2024). Reactive oxygen species and mitochondrial calcium’s roles in the development of atherosclerosis. Curr. Pharm. Des. 30, 1812–1821. doi: 10.2174/0113816128303026240514111200 38803178

[B28] Qu K. Yan F. Qin X. Zhang K. He W. Dong M. . (2022). Mitochondrial dysfunction in vascular endothelial cells and its role in atherosclerosis. Front. Physiol. 13, 1084604. doi: 10.3389/fphys.2022.1084604 36605901 PMC9807884

[B29] Ren H. Hu W. Jiang T. Yao Q. Qi Y. Huang K. (2024). Mechanical stress induced mitochondrial dysfunction in cardiovascular diseases: Novel mechanisms and therapeutic targets. Biomed. Pharmacother. 174, 116545. doi: 10.1016/j.biopha.2024.116545 38603884

[B30] Ridker P. M. Everett B. M. Thuren T. MacFadyen J. G. Chang W. H. Ballantyne C. . (2017). Antiinflammatory therapy with canakinumab for atherosclerotic disease. N. Engl. J. Med. 377, 1119–1131. doi: 10.1056/NEJMoa1707914 28845751

[B31] Salnikova D. Orekhova V. Grechko A. Starodubova A. Bezsonov E. Popkova T. . (2021). Mitochondrial dysfunction in vascular wall cells and its role in atherosclerosis. Int. J. Mol. Sci. 22, 8990. doi: 10.3390/ijms22168990 34445694 PMC8396504

[B32] Singh J. Stanley C. P. Kavurma M. M. (2026). Endothelial mitochondrial dysfunction in hypertension, diabetes, and atherosclerosis. Cardiovasc. Res. 122, 19–32. doi: 10.1093/cvr/cvaf282 41490203

[B33] Stachowicz A. Wiśniewska A. Czepiel K. Pomierny B. Skórkowska A. Kuśnierz-Cabala B. . (2024). Mitochondria-targeted hydrogen sulfide donor reduces atherogenesis by changing macrophage phenotypes and increasing UCP1 expression in vascular smooth muscle cells. Biomed. Pharmacother. 180, 117527. doi: 10.1016/j.biopha.2024.117527 39405912

[B34] Stamerra C. A. Di Giosia P. Giorgini P. Ferri C. Sukhorukov V. N. Sahebkar A. (2022). Mitochondrial dysfunction and cardiovascular disease: Pathophysiology and emerging therapies. Oxid. Med. Cell. Longev. 2022, 9530007. doi: 10.1155/2022/9530007 35958017 PMC9363184

[B35] Stepaniuk N. Stepaniuk A. Hudz N. Havryliuk I. (2024). The impact of mitochondrial dysfunction on the pathogenesis of atherosclerosis. Wiad. Lek. 77, 153–159. doi: 10.36740/WLek202401119 38431820

[B36] Su Z. D. Li C. Q. Wang H. W. Zheng M. M. Chen Q. W. (2023). Inhibition of DRP1-dependent mitochondrial fission by Mdivi-1 alleviates atherosclerosis through the modulation of M1 polarization. J. Transl. Med. 21, 427. doi: 10.1186/s12967-023-04270-9 37386574 PMC10311781

[B37] Wang Z. Sun W. Zhang K. Ke X. Wang Z. (2025). New insights into the relationship of mitochondrial metabolism and atherosclerosis. Cell. Signal. 127, 111580. doi: 10.1016/j.cellsig.2024.111580 39732307

[B38] Wang J. Zou J. Shi Y. Zeng N. Guo D. Wang H. . (2024). Traditional Chinese medicine and mitophagy: A novel approach for cardiovascular disease management. Phytomedicine 128, 155472. doi: 10.1016/j.phymed.2024.155472 38461630

[B39] Wu Q. Cai Y. Yan Z. Wang Y. Liu J. Guan X. . (2025). Redox-sensitive m(6)A regulation in atherosclerosis: METTL3 at the crossroads of mitochondrial quality control and pyroptosis. Redox Biol. 88, 103918. doi: 10.1016/j.redox.2025.103918 41240787 PMC12663850

[B40] Xia D. Liu Y. Wu P. Wei D. (2023). Current advances of mitochondrial dysfunction and cardiovascular disease and promising therapeutic strategies. Am. J. Pathol. 193, 1485–1500. doi: 10.1016/j.ajpath.2023.06.013 37481069

[B41] Xu M. Wang W. Cheng J. Qu H. Xu M. Wang L. (2024). Effects of mitochondrial dysfunction on cellular function: Role in atherosclerosis. Biomed. Pharmacother. 174, 116587. doi: 10.1016/j.biopha.2024.116587 38636397

[B42] Xu K. Wang L. Lv T. Jin C. Wu Q. Zhou Y. . (2025). Nanomedicines against mitochondrial dysfunction-induced metabolic diseases. Adv. Sci. (Weinh). 12, e14522. doi: 10.1002/advs.202514522 41268707 PMC12752600

[B43] Xue S. Su Z. Liu D. (2023). Immunometabolism and immune response regulate macrophage function in atherosclerosis. Ageing Res. Rev. 90, 101993. doi: 10.1016/j.arr.2023.101993 37379970

[B44] You B. Yi D. Wang B. Yan H. (2025). The complex bidirectional relationship between aging and atherosclerosis: Mechanistic insights and translational opportunities. Aging Dis. 17, 1223–1236. doi: 10.14336/AD.2025.10620 40681353 PMC13061554

[B45] Yu W. Wang X. Zhao J. Liu R. Liu J. Wang Z. . (2020). Stat2-Drp1 mediated mitochondrial mass increase is necessary for pro-inflammatory differentiation of macrophages. Redox Biol. 37, 101761. doi: 10.1016/j.redox.2020.101761 33080440 PMC7575803

[B46] Zakirov F. H. Zhang D. Grechko A. V. Wu W. K. Poznyak A. V. Orekhov A. N. (2020). Lipid-based gene delivery to macrophage mitochondria for atherosclerosis therapy. Pharmacol. Res. Perspect. 8, e00584. doi: 10.1002/prp2.584 32237116 PMC7111069

[B47] Zhang Z. Zhao X. Zhang X. Wan S. An P. Zhu Y. . (2026). Mitochondrial cardiovascular diseases: Molecular mechanisms, multi-omics exploration and therapeutic strategies. J. Adv. Res. 82, 347–365. doi: 10.1016/j.jare.2025.07.021 40680831 PMC13001046

[B48] Zheng L. Chen X. He X. Wei H. Li X. Tan Y. . (2025). METTL4-mediated mitochondrial DNA N6-methyldeoxyadenosine promoting macrophage inflammation and atherosclerosis. Circulation 151, 946–965. doi: 10.1161/CIRCULATIONAHA.124.069574 39687989 PMC11952693

[B49] Zhong Y. L. Xu C. Q. Li J. Liang Z. Q. Wang M. M. Ma C. . (2025). Mitochondrial dynamics and metabolism in macrophages for cardiovascular disease: A review. Phytomedicine 140, 156620. doi: 10.1016/j.phymed.2025.156620 40068296

[B50] Zhou J. Liu H. Ma S. Wang H. He N. Zhang X. . (2026). Mitophagy in macrophages: A metabolic checkpoint in inflammation-to-repair transition in atherosclerosis. J. Am. Heart Assoc. 15, e048103. doi: 10.1161/JAHA.125.048103 41902740 PMC13323179

